# On the influence of thought on language: a naturalistic framework for the pantomimic origins of human communication

**DOI:** 10.3389/fpsyg.2023.1197968

**Published:** 2023-05-31

**Authors:** Francesco Ferretti

**Affiliations:** Cosmic Lab, Department of Philosophy, Communication and Performing Arts, Roma Tre University, Rome, Italy

**Keywords:** language and thought, mental space travel, mental time travel, mindreading, narrative, origin of language, pantomime, storytelling

## Abstract

This paper focuses on the idea that pantomime is a privileged lens for investigating the origin of language in a naturalistic framework. Two reasons support this claim. The first one concerns the motivated and iconic character of pantomime compared to the arbitrary and abstract features of linguistic signs emphasized by the conventionalist thesis. The second reason is that a pantomimic account of language origin paves the way for a rethinking of the traditional hypothesis on the relationship between thought and language. Specifically, it leads to a revision of the thesis of the unidirectional influence of language on thought in favor of a bidirectional influence. Indeed, looking at the relationship between thought and language in its nascent stage means investigating the role of thought in shaping language rather than the role of language in shaping thought. A bidirectional perspective of this type hinges on the twofold idea that thought has primarily a narrative foundation and that pantomime represents an ideal expressive means for bootstrapping the evolutionary foundations of language origins in a naturalistic framework.

## 1. Introduction

At the basis of classical semiotics is the idea that the expressive code, besides being a means of communication, is a tool for shaping thought, hence, an important equipment of human mental faculties. Within theories inspired by conventionalism, the constitutive role of language on thought is widely ascribed to the abstract and arbitrary nature of the linguistic sign. Contrary to this tradition and consistent with the naturalistic conception, this paper offers an alternative viewpoint. Specifically, it is my claim that in the early stages of human communication thought and language are in a mirrored position with respect to each other compared to the claims of the conventionalist account. Indeed, looking at the relationship between thought and language in its nascent stage means investigating the role of thought in shaping language rather than the role of language (which is not there yet) in shaping thought. In claiming that the form of thought constrains language, I am arguing that the chance for our ancestors to use an expressive system able to foster language was affected by reasons of expressive efficiency. In a similar naturalistic perspective, pantomime could have represented a starting point for human communication in that it was a good candidate for conveying the form of thoughts, of which it reflects the constitutive structure. The assumption underlying this article, in fact, is that thought has a narrative structure and that pantomime represents the way invented by our ancestors to express the narrative form of thoughts in the absence of language.

## 2. A classical framework

A main issue of Western philosophy of language since its inception is establishing whether words refer to entities in the world by nature or by convention. In the ancient Greek, the debate was polarized between the idea that words reflect the essence of things and the hypothesis that words derive from established cultural conventions. The conventionalist thesis emphasizes the arbitrariness of the relationship between names and entities based on the lack of any intrinsic connection between them; the naturalistic conception challenges the arbitrary nature of the linguistic sign showing why the way in which words refer to things cannot be considered unmotivated.

The Plato’s *Cratylus* (Plato, 1997), which contributed to launch the Western philosophy of language, has at its center the dispute between conventionalism and naturalism. In the dialogue Cratylus defends the naturalistic perspective while Hermogenes favors the conventionalist thesis. In favor of his view, Cratylus holds that the accuracy of a name “indicates the thing as it is” (428e), whereas Hermogenes claims that nothing but convention determines the correctness of names since “no name belongs to any particular thing by nature, but only by the habit and custom of those who employ it and who established the usage” (384d-e). In the first part of the dialogue, Socrates (who expresses Plato’s theory) refutes conventionalism, arguing that the essence of things can constrain the designation of names. In favor of Cratylus, Socrates refers to sound symbolism, an explanatory tool which thus far is an effective way to control relativism connected to the conventionalist account of language origins.

The debate on the natural or conventional foundation of language has been central in the 17th and 18th Century philosophy. The controversy between Locke and Leibniz serves as an example of a novel aspect of the contemporary debate on the topic. In the *New Essays on Human Understanding* (Leibniz, 1765/2022), Leibniz rejects the arbitrary view of language supported by Locke in *An Essay Concerning Human Understanding* (Locke, 1689/1998). Within a naturalistic perspective, Leibniz accounts for the motivated character of names appealing to sound symbolism but with an important difference with respect to the classical thesis. In his view, indeed, the possibility of sounds to refer to entities of the world is driven by a psycho-emotional factor: the naturalness of the sign does not concern its relationship with the essence of things but rather with the internal affective state of the individual.

In spite of Leibniz’s critique, the Lockean thesis is still prevailing, mostly justified by the priority assigned to the arbitrariness of the linguistic sign: if words have no intrinsic connection with things but just with ideas, then language has a conventional origin. The Lockean notion of arbitrariness has been crucial within a tradition that, from Condillac to the French Ideologues and Humboldt, has accomplished its final (and radical) stage with De Saussure (1916) (for a discussion: Aarsleff, 1982), who defends the radical arbitrariness of the linguistic sign. The radical thesis of the arbitrariness of signs is a way to claim the systematic character of languages and to assign to the super-individual nature of the sign a sort of expressive solidity that cannot be justified through the mere reference to the arbitrary character of the sign. In fact, the novelty of Saussure’s approach lays in the way of considering the relationship of necessity between the two faces of the linguistic sign as emerging exclusively within a given code, once linguistic members have established to accept that relationship resting upon a social agreement. Each language is indeed a system of values autonomous from any other and the arbitrariness of the sign ensures order to language as a system of independent values deeply interconnected with each other (De Mauro, 1971). This order of considerations favored a conception of human beings founded on the idea that language is essential because it is not only a communication system, but it also gives shape to thought. An interpretative hypothesis based on similar arguments connects the conventionalist thesis with a strong form of linguistic relativism founded on the thesis of the constituent role of language on thought, namely the idea that mental content (the essential property of thoughts) is strictly dependent on language. In contrast with the conventionalist thesis, the present paper offers a naturalistic account of language origin founded on two main arguments: the first argument concerns the nature of the expressive system used by our ancestors in the early stages of linguistic communication; the second refers to the relationship between the expressive system and mental content in the initial phases of human communication.

## 3. Motivation and transparency of gestures

A classical argument in favor of the conventionalist theory hinges on the abstract and unmotivated character of linguistic signs. In this view, signs do not rest on sound symbolic precursors since the abstract and arbitrary features cannot be derived from the iconic and motivated character of sound symbolic expressions – in other terms, the emergence of symbolic signs is all but a qualitative leap. A reply to this argument involves the idea of a gradual transition from the iconic to the arbitrary ground, which does not necessarily entail a sharp gap between the two forms as most acts of sign are not arbitrary in absolute sense. For example, Zlatev (2008, 2014a,b) emphasizes that conventionality of semiotic codes, even in their most abstract forms, does not preclude some degree of iconicity (forms of sound symbolism). This applies a fortiori in the case of gestures: although gestures may reach a degree of arbitrariness quite similar to that of verbal signs, the evolutionary process “from the iconic to the abstract” cannot be interpreted in terms of a qualitative leap since the ubiquity of iconicity pertains to both uncoded and coded gestures (Poggi, 2008). In such a gradualist perspective, the transition from the iconic to the abstract ground proceeds through expressive forms characterized by the absence of *pure* traits one way or the other. The appropriate distinction is rather between “predominantly mimetic” and “predominantly symbolic” forms of communication (Zlatev, 2014b, p. 166). Therefore, the arbitrary and abstract character of linguistic signs cannot be considered as a prime example of a qualitative difference between mimetic and symbolic communication. In language evolution, the idea that the emergence of communication begins with iconic signs is not a hindrance to the evolution of arbitrary signs.

That said, the main strength of mimetic expressions is that they appear to be particularly advantageous in bootstrapping communication. In this regard, gestural communication is particularly relevant. Several scholars have highlighted the “transparency” of gestures in the absence of social conventions as in the early stages of communication (Arbib, 2012). The thesis of the transparent nature of gestures has been deeply validated by the discovery of mirror neurons. In a seminal paper, Rizzolatti and Arbib (1998) have claimed that gestures can convey transparent meanings since they are basically action frames and humans are endowed with a neural system for action recognition. As the mirror system generates internal simulations of another individual performing actions and that simulation process is a way to understand those actions, then the mirror system provides a suitable mechanism for the comprehension of gestural communication (Corballis, 2002, 2017; Rizzolatti and Sinigaglia, 2008). These considerations are relevant for evolutionary issues: in the absence of semiotic conventions, our predecessors could have used gestural forms of expressions.

The idea that the mirror system may account for gestural comprehension processes paves the way for the second issue to be addressed: the relationship between the expressive system and the content to be expressed. It is a crucial issue for the dispute between conventionalism and naturalism since it closely affects the topic of linguistic relativism, namely the topic of how to intend the relationship between language and thought.

## 4. Relativism

Relativism does not entail a unique hypothesis. An important distinction to be made is the difference between “strong” and “weak” forms of relativism. Strong relativism is perfectly represented by linguistic determinism, that is, the idea that language not only expresses but also determines thoughts; the weak version argues that language influences thought without determining it.

Linguistic determinism is traditionally associated with the controversial Sapir-Whorf hypothesis (Sapir, 1921; Whorf, 1956). However, the arguments underlying this hypothesis are generally rejected by current research. Many authors even conclude that it is inappropriate to ascribe to Whorf himself a radical position (cf. Gentner and Goldin-Meadow, 2003). For example, Geertz (1984) argued that a drift of attitude has spread using relativism as a specter to cast out, leading to a tilt at windmills.

Whatever Whorf may originally have said, today linguistic determinism is going through an impressive revision process. The prevailing idea among neo-Whorfian scholars is that such a process should result in the weakening of radical theses: after abandoning the hypothesis of a constitutive role of language over thought, the common thesis posits that language has simply *a role* in the formation of thought (“the Whorf-effect,” as named by Levinson, 2003). Everett (2012) participates in rethinking Whorfianism by arguing that the languages we speak can influence but not determine the way we think. In a similar vein, Hagoort (2023, p. 3) proposes the language marker hypothesis, stating that “our uniquely human language capacity provides *Homo sapiens* with additional machinery for the creation of rich internal models.”

The thesis of linguistic influence on thought is considered as a way to oppose the ideological drift of linguistic determinism (Zlatev and Blomberg, 2015; Blomberg and Zlatev, 2021). Such a thesis is particularly interesting for our purposes since the way of conceiving the relationship between language and thought is a key to investigating the issue of the natural or conventional character of language origin. In favor of the naturalistic approach, in the present work I propose a two-steps argument: first, I make use of the arguments underlying the thesis of linguistic influence in order to support the independence of thought from language; then I show that disentangling thought from language may also provide hints for the thesis of the influence of thought on language—a thesis entirely unexplored by neo-Whorfian scholars beyond a declaration of intent. This aspect is fundamental because, as much as the relationship between thought and language should be bidirectional, I claim that in the early stages of linguistic communication the path going from thought to language had an initial priority. It is only starting from the analysis of the influence of thought on language that it is possible to define the role of the gestural system (specifically, of the pantomimic system) within a naturalistic framework of language origin.

## 5. Language may influence thought

Against linguistic determinism, Zlatev and Blomberg (2015) and Blomberg and Zlatev (2021) claim that a necessary step toward the thesis of linguistic influence on thought is disentangling language from thought. The claim is entirely acceptable: it is impossible to argue for the influence of language on thought without acknowledging a form of autonomy of thought from language. But the key to understanding if Zlatev and Blomberg (2015) make a good step forward is considering the definition of thought they propose:

By “thought”, we mean essentially *mediated cognition*. This corresponds approximately to what are sometimes called “higher cognitive processes”, in which the mind is not fully immersed in the practical concerns of the here-and-now, but rather employs various structures and processes of conscious awareness such as mental imagery, episodic memories or explicit anticipations to focus on intentional objects that are not perceptually present (Zlatev and Blomberg, 2015, p. 2).

I do not question that mediated cognition is a distinctive trait of some psychological abilities engaged in language processing nor do I question that language may indeed influence cognition (cognitive modalities such as memory, reasoning, mental time travel, and so on). But I question the identification between cognition and thought. The authors interpret mediated cognition in terms of the folk-psychological concept of “thought” and “thinking.” It is likely that the two terms can be interchanged in common sense but, when it comes to a scientifically-grounded reflection, thinking and thought require to be distinguishable. To justify the thesis of linguistic influence, it is indeed essential distinguishing *how* we think (the way of thinking a given content) from *what* we think (the content that is possible to think in different ways). For example, I can maintain the thought of a cat eating kibble either by remembering my cat the day before or by imaging when my cat will ask for food at lunchtime or by looking at my cat eating in front of my eyes.

An important clarification is that in distinguishing thinking and thought I do not intend to suggest a metaphysical distinction for which the meaning of sentences is independent from how humans actually think (as proposed by Frege, 1918). There are no thoughts out of actual thinking. That said, at the center of reflection on the relationship between thought and language lies the topic of the content of thoughts more than the topic of how humans process that content. When claiming that language influences thought, indeed, that influence concerns what we think and not (or not exclusively) the way we think something. The classical thesis of linguistic determinism has taken a stand on the question by arguing that language limits and determines thought. In a similar way, the thesis of linguistic influence requires explaining how language influences the content of thoughts – how there can be a form of mental content before and independently of the influence of language over thought.

A further evidence that the thesis of linguistic influence does not raise (and should not raise) questions pertaining to the content of thoughts in the view of Blomberg and Zlatev (2021) comes from their dissociation from cognitive linguistics. In their view, the embodied perspective of the mind (deeply aligned with Merleau-Ponty’s phenomenology) can explain how language can influence thought:

First of all, acknowledging the existence of a universal embodied level of human experience on which all languages are grounded, undercuts the most extreme forms of linguistic relativity, such as those of the classical relativists who saw languages as incommensurable, and “arbitrary” with respect to one another. It is such, empirically as well as conceptually, unsupported claims that have led to extreme claims that each language imposes its own “reality”, i.e. linguistic determinism. The embodied level is necessary to be able to formulate the conceptual framework in which to compare languages, as acknowledged by Whorf. Since it is not a level of actual linguistic structures but of motivating processes, to this level should be attributed hypothetical notions like “image schema” and “conceptual metaphors” (see Zlatev, 2011), and “non-actual motion” (Blomberg, 2015), often studies in cognitive linguistics as part of linguistic meaning rather than as motivation for it, as we emphasize” (Blomberg and Zlatev, 2021, p. 44).

It is worth noting here that the constraints of the embodied level to the emergence of linguistic structures are only *motivations* that do not involve the content level conveyed by those structures (as explicitly acknowledged, it is exactly on this point that they distance themselves from cognitive linguistics). Nevertheless, the idea that motivations act as constraints to the linguistic structures, reasonable though it may be, taken by itself cannot justify the thesis of the linguistic influence on thought. Motivations are expedients for limiting the way of thinking (along with the way of expressing thoughts) and work perfectly against linguistic determinism; however, without referring to the content of thoughts, it is not possible to disentangle language and thought, thus, it is not possible to account for the thesis of linguistic influence. To this aim, a strong claim regarding the distinction between language and thought is required. In the neo-Whorfian hypothesis, a suitable notion of thought is lacking. The core of the problem is that the content of thoughts entails the notion of mental representation, which is strongly questioned by embodied theories inspired by phenomenology. It represents a core problem since:

- without a notion of mental representation, there can be no mental content;
- without any reference to mental content, there can be no thought;
- without thought, we cannot justify the thesis of linguistic influence on thought.

The important operation of disentangling language and thought paves the way for a second aspect to be considered in the analysis of the relationship between language and thought. Within the theoretical perspectives arguing for the thesis of influence, progression in such a relationship is unidirectional: it is language that affects thought and never the reverse. Disentangling thought from language at the content level means viewing that progression in a specular manner compared to the neo-Whorfian conception. As we will see, this is a critical theoretical move to account for language origin within a naturalistic framework.

## 6. The content of thoughts

In contemporary philosophy of the mind and cognitive science, the debate on the nature of thought relies on the thesis of mental states as propositional attitudes, in terms of relation between agents and representations. The traditional idea is that the content of thoughts is represented in the mind in sentence-like propositions of the Language of Thought (LoT; Fodor, 1975, 1987). In this view, without a reference to the propositional representation of content we cannot speak about thought in the proper sense.

The claim that the propositional form is the unique form of representation is a main tenet of the cognitive mainstream since its foundation. It has important implications for the issue of the relationship between language and thought: unlike those who support the thesis of the linguistic influence, Fodor posits that the constituent structure of language depends on the constituent structure of thought since language can express thought only by mirroring its form. Emphasizing the amodal and abstract features of representation, the account of propositional attitudes has been predominant when the study of the mind was based on the computer metaphor.

The representational model is no longer at the center of theoretical interests (see for a different point of view: Quilty-Dunn et al., 2022). Among the elements which undermined the LoT hypothesis, the critique of the amodal and abstract character of propositions guided by the formality condition and methodological solipsism has a prominent role. Criticisms of the classical model go beyond the purposes of the present paper. It is sufficient to underline that the LoT hypothesis cannot overcome the critique of linguistic idealism brought against the supporters of relativism (weak or strong it is). In fact, while it is true that in Fodor’s view thought determines the form of language, this translates into a substantial identity between language and thought with implications not dissimilar to those of linguistic determinism, though in a specular manner.

Before proceeding further, an issue requires to be highlighted. Many criticisms of the notion of mental representation made by the proponents of embodied cognition concern the way of intending representation in classical cognitive science. But their skepticism towards the notion of mental representation leaves the difficulty of explaining how language can influence thought, provided that there can be no thought without a reference to mental representation. How to overcome this impasse? An option is considering a different notion of mental representation compared to that proposed by classical computational models. While the criticism towards the amodal and abstract nature of mental propositions of the LoT is entirely acceptable, the idea of excluding the concept of mental representation in the account of mental content is indeed completely unsuitable. In other terms, abandoning the propositional model of thought does not mean (should not mean) abandoning the representational models of the mind.

In the light of these considerations, I intend to propose a model of mental representation able to overcome the weaknesses of the propositional thesis and to give sense to the notion of mental content. To this aim, we need a less abstract concept of representation than that characterizing mental propositions, a concept that should be able to define how real humans represent reality and experience. Contrary to the propositional thesis, here I endorse the hypothesis of a narrative form of thought, in line with the idea that humans represent reality and experience in a story-like fashion. This entails a twofold constructive implication: it allows to properly take into consideration the thesis of linguistic influence on thought (as a thesis affecting the content of thoughts) but also to look at the process involving the influence of thought over language in a naturalistic perspective. This represents a crucial implication when it comes to the bootstrapping conditions for the emergence of language. As for the origins, in fact, thought is not merely autonomous and comes first but is primarily the evolutionary precondition of language. Specifically, it is the evolutionary prerequisite of how humans could begin expressing thoughts in order to communicate with the other group’s members.

## 7. The discursive dimension of thought

Within a pivotal article on the topic, Bruner (1991) claimed that humans, unlike other animals, think of reality and experience through narrative forms of representation. Niles (1999) defined the individuals of our species as *Homo Narrans* and the idea that the ability to tell stories is the hallmark of humans is widely accepted among scholars coming from different research fields (e.g., Brooks, 1984; Hutto, 2007, 2008; Herman, 2013; McBride, 2014; Corballis, 2017; Ferretti, 2021, 2022). A suitable definition of narrative has been proposed by Hinyard and Kreuter (2007, p. 778): “any cohesive and coherent story with an identifiable beginning, middle, and end that provides information about scene, characters, and conflict, raises unanswered questions or unresolved conflict, and provides solution.” The idea underlying the thesis of the narrative foundation of thought is that narrative characterizes human thought as the ability to represent reality in a story-like form cannot be reduced to any other form of representation.

In spite of some consensus on general issues, the debate on the nature and origin of narrative thought is highly controversial. Again, the object of the discussion is the relationship between thought and language. The advocates of culturalist constructivism state that humans are very early engaged in narrative (conventional) practices and thoughts largely result from an internalization of processes through social apprenticeship (Bruner, 1990; Hutto, 2008). Hutto (2008, p. 27), for example, holds that “narratives are a distinctive and characteristic feature of human cultural niches, just as dams are for beavers.” By referring to narrative practices and the processes of acquiring them during ontogenesis, Hutto takes part of a well-established tradition led by Bruner, who considers stories allowing to interpret reality as the result of a tradition stored in the socio-cultural practices of the community. In Bruner’s perspective, “it is culture, not biology, that shapes human life and the human mind” (Bruner, 1990, p. 35).

Similar considerations pave the way for the idea that narrative representation is mainly the result of a process in which language plays a leading role. Scalise-Sugiyama (2001, 2005) is among the authors who have insisted on the primacy of language over thought, arguing that humans “could not have used stories as a means of exchanging information prior to the emergence of language” (Scalise-Sugiyama, 2001, p. 225). After emphasizing that no other expressive forms can be as powerful as narrative since only narrative can simulate the complexity of human experience, Scalise Sugiyama (2005, p. 191) restates the validity of the Language First Hypothesis suggesting that “without language, it is extremely difficult to accurately represent characters, objects, goals, or obstacles.”

In such a perspective, in which language (specifically, verbal language) is the constitutive condition of narrative representation, it appears difficult to distinguish what depends on language from what depends on narrative. Even though, at first sight, the thesis of internalization of narrative practices seems to be in line with the idea of the linguistic influence on thought, this compliance is only apparent. In fact, if the thesis of the linguistic influence requires disentangling language and thought, then considering language as the constitutive structure of narrative thought seems to undermine at the root the idea of thought as distinct from language (for details: Ferretti, 2022). The thesis of the autonomy of narrative from language should therefore take a different direction compared to the perspective of the Language First Hypothesis. In line with a naturalistic approach, I propose a two-steps argument: first, I support the independence of narrative representation from language; second, I claim that the Language First Hypothesis should make room for the Narrative First Hypothesis in order to explain how narrative representation can influence language.

A way to give substance to the first argument is describing the existence of narrative thought independently from language. Against Bruner, I argue for the key role of biology (in particular, the brain and the cognitive architectures) along with culture in shaping the mind and human thoughts. In this view, understanding how there can be a narrative representation in the absence of language passes through the analysis of the neural and cognitive systems underlying a narrative brain: these systems are primarily involved in the narrative representation of reality characterizing the specific human way of thinking before and independently from language (Storr, 2019; Armstrong, 2020; Ferretti and Adornetti, 2020; Ferretti, 2022). As the possibility to distinguish between narrative and language relies on the existence of such systems, the investigation of the cognitive architectures at the basis of narrative processing deserves great attention.

## 8. The narrative brain

The idea that we can have narrative representations of reality independently from language hinges on the idea that the human brain is endowed with systems able to process the constitutive properties of stories. Although the issue of what defines stories is controversial, there is consensus that time, plot and character are among the constitutive features of narrative. These features, representing the extended dimension of stories, rely on specific properties and processing systems. Plot, for example, is driven by global coherence, which governs the organization of the causal sequence of events in time (Giora, 1985, 2014; Adornetti, 2015; Ferretti, 2022). Empirical investigations have supported the role of systems of spatio-temporal projection in the elaboration of global coherence (e.g., Race et al., 2015; Ferretti et al., 2018). By emphasizing the role of character (Fludernik, 1996), other studies have shown the impact of mindreading in the processing of stories (Brown et al., 2019). For example, the ability to detach from oneself to be in the character’s shoes underlies narrative transportation, a main characteristic of narrative comprehension (Gerrig, 1993; Green and Brock, 2000).

On the basis of these considerations, it is possible considering the systems of projection in space and time along with mindreading as main candidates for the processing of the extended dimension of narrative representation. More specifically, the narrative brain is governed by the Triadic System of Grounding and Projection (TSGP) (Ferretti, 2022), a functional macrosystem composed of Mental Time Travel, Mental Space Travel and Mental Mind Travel, which allows flexible behaviors through grounding processes governed by the detachment from the here-and-now. Even the simple perception of a scene is indeed a social-spatio-temporal extension of the represented scene: looking at an object, I can imagine its future use or remember a past use or even how and why that object has been used or will be used by other people. Perceiving something is always perceiving it in the wider context of the extended dimension of experience. If the extended dimension of experience is what defines narrative representation of reality, then the projective systems underlying TSGP can be viewed as the cognitive prerequisite to build this type of representations (Ferretti, 2022).

At the basis of the Narrative First Hypothesis is the idea that the systems comprising TSGP can construct a narrative (proto-narrative, as we will see below) representation of reality before the emergence of language. In this perspective, the adaptive value of stories is primarily cognitive and only later communicative: the human ability to share thoughts by means of stories is dependent on the ability to represent reality in the form of stories. The cognitive impact of a representation extended in space, time and social context provides a great adaptive value to the individuals who can have it. Gottschall (2012) highlights the adaptive role of narrative viewing stories as tools that allow humans to experience situations without any of the risks they ordinarily involve, as in a flight simulator. With explicit reference to Aristotle, Corballis (2017) holds that stories allow imaging alternative worlds, paving the way for counterfactual reasoning, one of the most complex human cognitive capacities. Emphasizing that stories are means of knowledge is a way to acknowledge that narrative representation is evolutionarily driven by selective pressures not directly connected with the communicative function.

The reference to the narrative representation of reality resulting from the functioning of TSGP permits to give body to the idea of the independence of thought from language, disentangling the two entities. The existence of narrative representations autonomous from language opens the way to an aspect of the relationship between thought and language that is usually omitted in the thesis of the linguistic influence: the fact that thought can act as a constraint on language exactly as language does on thought. From this point of view, narrative representations are the joint product of the functioning of cognitive systems and of the restructuring of the resulting narrative content at the hands of language. To distinguish between the two forms of representation, I use *proto-narrative representation* to refer to that produced by the systems comprising the narrative brain and *narrative representation* to refer to the representation of reality influenced by language (Ferretti, 2022). In light of this distinction, it is possible clarifying the mutual influence between thought and language: if language influences the proto-narrative representations resulting from TSGP, the proto-narrative representations constrain the form that language may take on to effectively convey thoughts. The thesis of the mutual influence between thought and language introduces the possibility to address the issue of language origin within a naturalistic perspective: only with reference to the constraint posed by thought on language, indeed, the motivated nature of the expressive system can be justified. Against this naturalistic backdrop, the idea of a pantomimic foundation of language takes shape.

## 9. Pantomime as storytelling without language

On the basis of the above considerations, arguing for narrative as a distinctive trait of thought is a way for disentangling thought and language, but above all for establishing a reciprocal connection between them. Specifically, it is a way for taking into account the impact of thought on language in the early stages of human communication. Since the way of conveying content is subject to needs of expressive efficacy, in fact, it is likely that the first forms of communication were motivated by the form of the content to be expressed. If humans think of reality in the form of stories, then the way of conveying stories should depend on the type of thoughts to be expressed. In this view, the constraint posed by the nature of representations on the expressive system overrides any influence of language on thought. Against the conventionalist thesis, it is therefore possible to conceive the initial stage of human communication in naturalistic terms.

That said, if our ancestors could represent reality in a narrative form, how could they share with others their story-like thoughts? Pantomime represents an effective candidate for expressing stories in the absence of language (see Sibierska, 2017). Contrary to Scalise Sugiyama’s thesis, which considers stories as dependent on verbal language, it is possible to tell stories through bodily mimesis. In this regard, McBride (2014, p. 3) claims that “mimes come into being as a way of telling stories long before any possibility of language existed or was even anticipated.” Referring to bodily mimesis is a way of relying on a long tradition of thought that leads to Aristotle. Our ancient predecessors (probably *Homo ergaster*) began communicating their narrative thoughts to others by means of pantomime (Donald, 1991; Corballis, 2017). A definition of pantomime which goes in the direction of supporting this scenario has been proposed by McNeill (2000, p. 5): pantomime is a “dumb show, a gesture or a sequence of gestures conveying a narrative line, with a story to tell, produced without speech” (see for a more articulate definition of pantomime: Żywiczyński et al., 2018; Zlatev et al., 2020). In his interpretive model (which considers gesture and word as intertwined from the very beginning), gesture plays a prominent role in storytelling. In general, as Cassell and McNeill (1991, p. 375) write, the argument is that “when we add gesture to speech, we shed light on many of the same questions that have been the focus of attention by narratologists.”

When putting together the structural constraints posed by the cognitive architectures on content representation and the selective pressures in favor of a narrative form of communication, pantomime appears to be a good candidate for investigating language origins. However, in the same way as argued for the proto-narrative thought resulting from TSGP, also in this case a distinction between a proto-narrative communication characterizing pantomimic storytelling and a narrative communication made possible by verbal language is required. Pantomime can be considered a precursor of language since it represents the link between a proto-narrative thought and a narrative language (Ferretti, 2022; Ferretti et al., 2022).

The proto-narrative character of pantomime does not undermine its expressive capacity in terms of storytelling. Pantomime is indeed a plausible protolanguage because it contributes to building the narrative structure (the specific and distinctive trait) of modern verbal language (Ferretti, 2022). Differently from McNeill (2012), who considers pantomime as an evolutionary dead-end, it can actually be considered as an effective tool for sharing the mental stories underlying human thoughts. In this way, pantomime is an ideal protolanguage which can convey the flow of events governed by global coherence, the feature characterizing the basic structure of both the gestural and verbal forms of language.

In fact, to be critical for evaluating if pantomime is an evolutionary precondition of modern language is the issue of expressive efficacy. Traditionally, the transition from gesture to speech is viewed as a leap between two different expressive modalities, in which the selective pressures are driven by *what is lacking* in pantomime to constitute a fully-fledged language. Notwithstanding, this way of framing the question is consistent with the conventionalist approach which considers modern language as a system containing a level of arbitrariness and symbolic abstractness completely absent in the mimetic expressions. I have already mentioned the reasons why the transition from the iconic to the symbolic ground should not be considered in absolute terms. That said, in my perspective, it is the strength of pantomime (rather than its inadequacy) to be relevant for the evolutionary scenario; its strength is linked to its capacity for storytelling as pantomime is able to express the extended dimension of thoughts without language. This makes pantomime a crucial step towards the construction of modern language: the narrative structure of pantomimic proto-stories might be the scaffolding of language inherited by verbalization, and then incorporated and reconstructed by it, but never completely replaced.

Overall, this pantomimic scenario of language evolution fits with a naturalistic perspective on the origins of communication guided by the coevolution of language and thought. This represents a thesis of the linguistic influence on thought which has at its basis the thesis of the influence of thought on language.

## 10. Conclusion

The main claim of this paper is that pantomime is a privileged lens for investigating the origin of language in a naturalistic fashion. Two reasons support this claim. The first reason concerns the motivated and iconic character of pantomime compared to the arbitrary and abstract features emphasized by the conventionalist thesis. The second reason is that the pantomimic account of language origin paves the way for a rethinking of the traditional hypothesis on the relationship between thought and language. Specifically, it leads to a revision of the thesis of the linguistic influence on thought in favor of a bidirectional influence. A naturalistic perspective on the origin of language is, indeed, plausible only through the introduction of a notion of mental content able to account for the mutual influence of thought and language. While in the early stages of human communication the (proto-narrative) thought constrains pantomimic communication, the advent of verbal language (typical of *Homo sapiens*) marks a change of direction in which language chiefly influences thought. There can be a linguistic influence on thought only assuming a preexisting linguistic code which is, however, a late product of evolution. These considerations suggest that language can influence thought provided that a coevolutionary relationship takes place in which thought primarily influences language, with pantomimic storytelling acting as a possible bootstrapping for the evolution of complex verbal forms of communication.

## Data availability statement

The original contributions presented in the study are included in the article/supplementary material, further inquiries can be directed to the corresponding author.

## Author contributions

The author confirms being the sole contributor of this work and has approved it for publication.

## Funding

Funding for Open Access provided by the Department of Philosophy, Communication and Performing Arts, Roma Tre University.

## Conflict of interest

The author declares that the research was conducted in the absence of any commercial or financial relationships that could be construed as a potential conflict of interest.

## Publisher’s note

All claims expressed in this article are solely those of the authors and do not necessarily represent those of their affiliated organizations, or those of the publisher, the editors and the reviewers. Any product that may be evaluated in this article, or claim that may be made by its manufacturer, is not guaranteed or endorsed by the publisher.
